# Discovery of small molecule inhibitors of *Leishmania braziliensis* Hsp90 chaperone

**DOI:** 10.1080/14756366.2020.1726342

**Published:** 2020-02-12

**Authors:** Fernanda A. H. Batista, Sérgio L. Ramos, Giusy Tassone, Andrei Leitão, Carlos A. Montanari, Maurizio Botta, Mattia Mori, Júlio C. Borges

**Affiliations:** a São Carlos Institute of Chemistry (IQSC), University of São Paulo (USP), São Carlos, Brazil; b Department of Biotechnology, Chemistry and Pharmacy - Department of Excellence 2018-2022, University of Siena, Siena, Italy; c Medicinal Chemistry Group (NEQUIMED), IQSC-USP, University of São Paulo, São Carlos, Brazil; d Lead Discovery Siena S.r., Siena, Italy; e Sbarro Institute for Cancer Research and Molecular Medicine, Center for Biotechnology, College of Science and Technology, Temple University, Philadelphia, PA, USA

**Keywords:** Hsp90, inhibitors, fluorescence, leishmaniasis, molecular modelling

## Abstract

Leishmaniasis is a neglected disease caused by the protozoa *Leishmania ssp*. Environmental differences found by the parasites in the vector and the host are translated into cellular stress, leading to the production of heat shock proteins (Hsp). These are molecular chaperones involved in the folding of nascent proteins as well as in the regulation of gene expression, signalling events and proteostasis. Since *Leishmania spp*. use Hsp90 to trigger important transitions between their different stages of the life cycle, this protein family becomes a profitable target in anti-parasite drug discovery. In this work, we implemented a multidisciplinary strategy coupling molecular modelling with *in vitro* assays to identify small molecules able to inhibit Hsp90 from *L. braziliensis* (LbHsp90). Overall, we identified some compounds able to kill the promastigote form of the *L. braziliensis*, and to inhibit LbHsp90 ATPase activity.

## Introduction

Therapeutic regimens for the treatment of neglected tropical diseases (NTDs) are limited, have high toxicity, restrictions on use by some patients, and are difficult to adhere because they generally require parenteral and long-term administration. This scenario becomes even more complex given the low investment in research and development aimed at treating NTDs^1^.

The aetiological agents of NTDs may be protozoa, bacteria, helminths and viruses. Specifically, leishmaniasis is a neglected disease endemic in 98 countries that is caused by the protozoa *Leishmania ssp*., whose transmission in humans occurs through the bite of an insect belonging to the subfamily *Phlebotominae*^2^. According to the World Health Organization, leishmaniasis causes every year more than one million new cases and 20,000–30,000 deaths worldwide^2^^,^^3^. Current drugs approved for the treatment of leishmaniasis include pentavalent antimony, Amphotericin B, Miltefosine, Paromomycin and, in some specific cases, ketoconazole, itraconazole and fluconazole^2^. However, an increasing number of cases of acquired resistance to these drugs, coupled with toxicity, the lack of efficacy, and the need for parenteral administration (except Miltefosine) highlight the need of novel treatment options for leishmaniasis^2^^,^^4^.

The environmental differences found by the parasites from their existence in the insect vector until the mammalian host are translated into a form of cellular stress, leading to the production of heat shock proteins (Hsp)^5^. Indeed, for some parasites thermal stress is one of the signals responsible for cell differentiation to which the parasite must resist for adapting to the new environment^6^^,^^7^. Among the Hsp family, Hsp90s are evolutionarily conserved ATP-dependent chaperones that participate in the stabilisation and enhancement of different client proteins, many of which are essential for constitutive cell signalling and adaptive stress response^8^^,^^9^. Hsp90 chaperone function is not limited to assist the folding of nascent proteins, but extends to the regulation of gene expression, signalling events and proteostasis^7^^,^^10^. The ability of Hsp90 to affect important cellular transformations is greatly explored by intracellular protozoa such as *Trypanosoma*, *Leishmania*, *Toxoplasma*, and *Plasmodium spp*., which use Hsp90 to trigger important transitions between their different life-cycle stages^7^^,^^11^. Accordingly, Hsp90 is a multi-potential target in anti-parasite drug discovery and development^12–15^.

It is estimated that Hsp90 constitutes 2.8% of the total protein content of *L. donovani* promastigotes, and that the levels of this proteins increase in parasites subjected to thermal stress^16^. It was also indicated that Hsp90 from *L. major* (LmHsp90) plays a key role in the differentiation from the amastigote to the promastigote stage. Treatment of promastigotes with the Hsp90 inhibitor geldanamycin (GA) have induced the synthesis of specific proteins of the amastigote form in the promastigotes^17^^,^^18^. In addition, morphological changes of the parasites under treatment with GA have been very similar to those induced by thermal shock, which is also capable of inducing differentiation^17^. These data suggest that modulation of Hsp90 activity by heat shock-induced sequestration, or Hsp90 inhibition by GA, triggers the differentiation between the different stages of the parasite, thus highlighting the relevance of this protein in measuring environmental changes and controlling parasite growth^19^.

Based on above evidences, in this work we focussed our efforts in the identification of small molecule inhibitors of Hsp90 from *L. braziliensis* (LbHsp90). To this aim, we established a multidisciplinary approach combining molecular modelling with *in vitro* evaluations. Starting from a two-step structure-based virtual screening, 28 chemically diverse compounds were selected and tested *in vitro* for their ability to bind and inhibit recombinant LbHsp90, as well as to impair parasite growth and replication. Overall, three molecules were highlighted in this study based on their ability to kill the promastigote form of *L. braziliensis*, and to inhibit the ATPase activity of LbHsp90 at low micromolar concentration, thus becoming worth of further investigations as anti-leishmaniasis candidates.

## Materials and methods

### Homology modelling

Sequences of LbHsp90 and LmHsp90 N-terminal domain (LbHsp90N and LmHsp90, respectively) were retrieved from the UniProt Knowledgebase (UniProtKB – http://www.uniprot.org/) under the accession codes A4HL70 and Q4Q4I6, respectively, and were aligned using Clustal X^20^, obtaining 94% of sequence identity (supplementary Figure S1). The crystallographic structure of LmHsp90N in complex with ADP (PDB ID 3U67^21^) was selected as the structural template to model the LbHsp90N. The 3 D model of LbHsp90N was obtained using the Prime software, version 3.5^22^, by keeping ADP and three conserved water molecules (thereafter referred as W1, W2, and W3) in the model. The homology model was optimised through energy minimisation in explicit solvent by means of AMBER12 programme^23^. The ff12SB and GAFF force fields were used for the protein and the small molecule, respectively. In details, LbHsp90N/ADP complex was solved in a rectilinear box of TIP3P3 water molecules buffering 6 Å from the macromolecular system. Energy minimisation was performed in two consecutive steps: (i) the solvent alone was energy minimised for 1000 steps with the Steepest Descent algorithm (SD) and subsequent 200 steps with the Conjugate Gradient algorithm (CG); (ii) the whole solvated complex was energy minimised for 1500 steps SD and subsequent 6000 steps CG. Pymol software (The PyMOL Molecular Graphics System, version 1.7.1.0, Schrödinger, LLC) was used to visualise the model and to prepare graphics.

### First-round virtual screening

A multi-step structure-based virtual screening composed of an initial similarity search with ROCS (Rapid Overlay of Chemical Structures, OpenEye software)^24^, followed by molecular docking, visual inspection, and chemical diversity analysis was carried out. The ROCS query was generated on the binding conformation of ADP in the relaxed homology model of LbHsp90N (supplementary Figure S2) by means of the vROCS graphic interface (OpenEye, Santa Fe, NM)^25^. The MolPort database (MolPort – https://www.molport.com/shop/index), consisting of around six million commercially available compounds, was screened in this work. Ligand conformational analysis was performed with OMEGA version 2.5.1.4 (OpenEye, Santa Fe, NM)^26^ generating up to 600 conformers for each molecule. Default parameters were used, except for *strictstereo* that was set as ‘false’, and *fromCT* that was set to ‘true’.

The MolPort database was then screened on the query using ROCS^25^. A total of 66,294 compounds endowed with a Tanimoto Combo value greater than 0.8 (arbitrary threshold value) were retained, and were docked towards the LbHsp90N homology model. The receptor for molecular docking was prepared by the *make_receptor* utility of OEDoking (OpenEye, Santa Fe, NM) version 3.0.0, with default settings. The potential shape was identified by molecular probes, and a constraint on Asp78 as hydrogen bond donor was set. Receptor box volume was 10720 Å^3^ with the dimensions of 24.00 * 23.33 * 20.00 Å. Molecular docking was performed with FRED docking programme (OpenEye, Santa Fe, NM)^27^, setting the resolution value as High.

### Second-round virtual screening

The moieties of Glb08 and Glb11 that were found to interact with Asp78 and W1, W2, and W3 of LbHsp90N were drawn in MarvinSketch software (ChemAxon – https://chemaxon.com/) (supplementary Figure S3) and were used to generate in the corresponding SMARTS notation of the substructure of Glb08 and Glb11, namely [H]N = C(N([H][H])N([H])[$([#1,*])] and [H]N = C(S[$([#1,*])]N([H])[H], respectively. The Ligand Filtering tool of LigPrep (Schrödinger Maestro suite)^28^ was used to filter the MolPort database, using the above SMARTS notations as queries. Overall, 2348 compounds were filtered and moved to the further docking study. Ligand energy minimisation was carried out by Szybki version 1.8.0.1 (OpenEye Scientific Software, Santa Fe, NM)^29^, with the MMFF94S force field and default parameters, while the most probable ionisation form of each molecule at pH 7.4 was generated by Fixpka (OpenEye Scientific Software, Santa Fe, NM)^30^. Conformational analysis and molecular docking were performed by using the same procedure already described above. Finally, the binding mode of 15% top ranking compounds was visually analysed.

### Protein expression and purification

The LbHsp90 and LbHsp90N (amino acid residues 1–221) recombinant proteins were expressed and purified as previously described^31^. Hsp90β and its N-terminal domain construct (Hsp90βN – residues 1–223) were produced as described in Minari et al.^32^. These plasmids were used to transform cells of *Escherichia coli* BL21(DE3) strain, which were grown in LB medium at 37 °C until reaching an OD_600 nm_ about 0.6–0.8, in the presence of the appropriate antibiotic. Protein expression was induced by the addition of 0.4 mM IPTG, and kept at constant temperature for 18 h at 18 °C for hHsp90β, and 4 h at 37 °C for hHsp90βN. Induced cells were then harvested by centrifugation, and the bacterial pellet was disrupted by sonication in 20 mM sodium phosphate (pH 7.4), 20 mM imidazole, and 500 mM NaCl (20 mL of buffer/L of culture medium), after incubation with 5 U of DNAse and 30 μg/mL of lysozyme for 40 min on ice. The supernatant of the lysed cells, obtained by centrifugation at 11,000 rpm for 30 min at 4 °C, was filtered using a 0.45 μm membrane filter and subjected to protein purification protocol as described for LbHsp90 recombinant protein^31^. Both N-terminal constructions were incubated with 1 U of thrombin/mg of protein for 12–14 h at 4 °C for His-tag cleavage. The purification efficacy was attested by 12% SDS-PAGE. Proteins concentrations were spectroscopically determined (at 280 nm) using the molar extinction coefficient predicted by the protein amino acid sequences at water conditions.

### Interaction screening by differential scanning fluorimetry

The interaction of Hsp90 proteins with different compounds was monitored through the melting temperature (*T_m_*) of each protein in the presence of a fixed concentration of the target compound by differential scanning fluorimetry (DSF) also referred as thermal shift assay^33^. A final concentration of 10 µM of dimeric LbHsp90 and 20 µM of monomeric LbHsp90N in 40 mM Hepes buffer (pH 7.5) containing 100 mM KCl were used. The DMSO concentration in each sample was fixed at 2.5% (v/v). Prior to the readings, the samples were incubated for 30 min on ice. The fluorophore Sypro Orange (Life Technologies) was used as fluorescent probe. The experiments were performed at a thermocycler CFX96 Touch Real Time PCR Detection System (BioRad) and the data were analysed using the CFX Manager software (BioRad). As positive controls, 100 µM of GA and Radicicol (RDC) were used.

### Cell viability assay

The leishmanicidal activity of the compounds was tested *in vitro* against the promastigote form of *L. braziliensis* applying 200 μM/well, (with a total of 0.5% DMSO) into 90 μL of culture. The concentration of *L. braziliensis* (MHOM/BR/1973/M2269) was adjusted to 10^7^ cells/mL. Amphotericin (100 μM/well) was used as positive control, and 0.5% DMSO as negative control.

The plates were incubated at room temperature (RT) for 72 h. After this period, the viability of the promastigotes was verified via MTT (3–(4, 5-Dimethylthiazol-2-yl)-2, 5-Diphenyltetrazolium Bromide) colorimetric assay. MTT/PMS 11.1 μL/well solution (MTT = 5 mg/mL; Phenazine methosulfate-PMS = 0.22 mg/mL) was added, and the plate was incubated at RT for 4 h, at 150 RPM, protected from light. Formazan crystals were then solubilised by adding 100 μL/well of a solubilisation solution containing 7% SDS, 10 mM HCl and 30% DMSO. The mixture was incubated at RT for 90 min at 150 RPM protected from light. Wells containing the medium and the compounds or DMSO without MTT were used as blank controls. The absorbance was quantified in a plate reader (Biotek Synergy HT) at 570 nm.

The cell viability percentages were calculated by the following equation:

(1) $$\mathrm{Cell}\;\mathrm{viability}\;\left(\%\right)=\;\left(A_{T}-A_{B}\right)/\left(A_{U}-A_{B}\right)\;\times 100$$

where *A_T_* was an averaged absorbance from treated samples, *A_B_* blank absorbance and *A_U_* was an averaged absorbance from negative control.

### Cytotoxic assay

Compounds cytotoxicity was evaluated in 100 μL of Balb-c 3T3 cone A31 mouse fibroblasts (10^5^ cells/mL) into a 96-well plate, incubated for 24 h at 37 °C to become adhered. After this period, the medium was washed and 100 μL of each compound was added to the culture medium (concentration ranging from 1 to 200 μM/well, with a total of 0.5% DMSO) in quadruplicate. Rapamycin was used as a positive control, and 0.5% DMSO was used as negative control. The plates were incubated for 72 h at 37 °C. After this, the viability of Balb-c 3T3 cells was evaluated by MTT colorimetric assay, as described previously.

### In vitro IC*_50_ determination*

For determination of the inhibitory concentration able of killing 50% of the parasites (*IC*_50_), an aliquot of 10 μL of each compound (concentration ranging from 1 to 200 μM/well, with a total of 0.5% DMSO) was added in quadruplicate on a 96-well plate containing 90 μL of culture with the promastigote forms of *L. braziliensis*, previously adjusted to 10^7^ cells/mL. Amphotericin 100 μM/well and 0.5% DMSO were used as positive and negative control, respectively. The plates were incubated at RT for 72 h. After this, the viability of the promastigotes was verified by MTT/PMS colorimetric assay, as described above. *In vitro* leishmanicidal activity, expressed as *IC*_50_, was determined using GraphPad Prism 5 software according to the following equation:

(2) $$y=\;A_{\min}\;+\;\left(A_{\max}-A_{\min}\right)/\left({1+10}^{\left(\left(\log{\mathrm{IC}}_{50}\;-x\right)*\mathrm{Hillslope}\right)}\right)$$

where *A_min_* and *A_max_* are, respectively, the minimum and maximum activities obtained, *x* is the ligand concentration and Hill Slope is the parameter that describes the steepness of the curve.

The selectivity index parameter was obtained by the ratio of compound *IC*_50_ against mammalian cells and compound *IC*_50_ against promastigote cells.

### *In vitro* interaction assay

Two µM of protein in 40 mM Hepes buffer (pH 7.5), 100 mM KCl, was incubated for 30 min on ice with increasing concentrations of the compounds diluted in DMSO. The DMSO concentration did not exceed 2% of the total reaction volume (v/v). The samples were excited at 280 nm and the emission was collected in the 300–400 nm range, in a 1.0 × 0.2 cm optical path cuvette, on an F-4500 Fluorescence Spectrophotometer (Hitachi). The fluorescence quenching was analysed at the maximum emission wavelength in the absence of ligand. The determination of the apparent dissociation constant (*K*_Dapp_) was performed by fitting the fluorescence values obtained for each compound concentration according to a dose-response model through the Equation (3):

(3) $$y\;=\;F_{\min}\;+\;\left(F_{\max}-F_{\min}\right)/\left(1+10^{\left(\log K_{D}-\;[\mathrm{ligand}]\right)}\right)$$

where *F_min_* and *F_max_* are the minimum and maximum fluorescence intensity and [*ligand*] is the compound concentration used in the assay, according to the GraphPad Prism 5 software. The fluorescence intensities were corrected by inner filter effects as previously described^31^.

### Spectrophotometric ATPase activity assays

The ATPase activity of LbHsp90 and hHsp90β was evaluated spectrophotometrically through inorganic phosphate (P_i_) quantification following ATP hydrolysis, by the EnzChek™ assay kit (Invitrogen^®^) as previous described^31^^,^^34^. Briefly, a 2 µM solution of LbHsp90 and hHsp90β enzymes was prepared in 150 mM NaCl, 5 mM MgCl_2_ and 20 mM Tris-HCl, pH 7.5, and incubated with ATP (concentration range 0–5 mM) for 60 min at 37 °C. Then, the chromogenic substrate 2-amino-6-mercapto 7-methyl purine riboside (MESG) and the purine nucleoside phosphorylase (PNP) were added and the resulting reaction mixture was incubated for 30 min at 37 °C. The assays were performed in a 96-well plate and kinetic parameters determined by Michaelis–Menten fitting using GraphPad Prism 6 software.

### ATPase activity inhibition assays

The inhibitory activity against LbHsp90 and hHsp90β of compounds showing interaction in DSF assays (namely Glb08, Glb15, Gl23, Glb25 and Glb27) was determined using spectrophotometric ATPase activity assays as described above. Initial screenings were performed using 50 μM compound concentration in a reaction mixture consisting of 2 μM enzyme and 2.5 mM ATP. After 1 h incubation at 37 °C, MESG and PNP were added. The amount of P_i_ was estimated after 30 min incubation at 37 °C. GA was used as reference compound, reporting 100% ATPase activity inhibition (at 50 μM). Compounds having percentage inhibition higher than 80% (namely compounds Glb08, Glb15 and Glb23 towards LbHsp90) were selected for *IC*_50_ determination. A compound concentration ranging from 1 to 75 μM was included in the reaction mixture and P_i_ quantification performed as previously described (measurements were performed in triplicate). *IC*_50_ values were determined by non-linear regression data fitting (using GraphPad Prism 6 software).

## Results

### Homology modelling

The lack of structural details on LbHsp90N seriously limited the rational design of specific small molecule inhibitors. We thus generated a homology model thanks to the availability of high quality structural templates from *L. major*, i.e. the LmHsp90N that shares 94% sequence identity with LbHsp90N, particularly within the ATP binding site (supplementary Figure S1). The structure of LmHsp90N in complex with ADP (PDB ID 3U67^21^) was selected as template in homology modelling, because it describes an almost physiological state of the parasite’s Hsp90. Structurally conserved water molecules W1, W2 and W3 were kept in the homology model because they are known from multiple Hsp90/ligand complexes to be crucial in mediating the interactions of the ADP, particularly with Asp78^35^. Active site composition of the two proteins differs at position 63, where isoleucine of LmHsp90N is replaced by valine in LbHsp90N. Although these residues share similar physicochemical features, they differ slightly in steric hindrance. Thus, energy minimisation in explicit solvent was performed to relax the homology model and to account for the Ile/Val replacement near the ligand binding site. In the relaxed model, the adenine moiety of ADP establishes a hydrogen bond with the carboxylate ion of Asp78, and is further involved in water-mediated interactions with the same Asp78, Leu33, and Gly82. The ADP ligand establishes additional H-bonds with Asn36, Asn91, Gly122, and Phe123. Overall, the LbHsp90N homology model was structurally similar to the template (Cα RMSD = 0.420 Å, supplementary Figure S2) as expected by the very high sequence identity between the two proteins (supplementary Figure S4), and it was subsequently used for structure-based molecular modelling studies.

### First-round virtual screening

The 3D representation of chemical features that are relevant in ligand/protein interaction is a powerful tool to screen *in silico* large chemical libraries. In this work, a pharmacophore-like ROCS query was generated on the binding mode of ADP into the homology model of LbHsp90N (supplementary Figure S3), and was used to filter the MolPort database. Molecules with a Tanimoto Combo value higher than 0.8 (around 60,000 molecules that correspond to 1.2% of the initial database) were selected for molecular docking studies towards the LbHsp90N homology model. The first 500 top-scoring compounds were visually inspected and further analysed according to multiple parameters, such as chemical diversity, scoring values, and visual inspection of the docking pose. Finally, 13 molecules (namely Glb01–Glb13, supplementary Figure S4), which are different from known Hsp90 inhibitors^15^, were selected and submitted to *in vitro* assays.

### Second-round virtual screening

To obtain insights into the structure-activity relationships of compounds identified in the first-round of virtual screening (see below), the scaffold of active compounds Glb08 and Glb11 was further expanded through a substructure search. Indeed, analysis of the docking-based binding modes of Glb08 and Glb11 highlighted two substructure patterns that account for the interaction with Asp78 and W1, W2, and W3. These are a guanidine in Glb08, and an imidothiocarbamate in Glb11 (supplementary Figure S4). Then, 2,348 compounds bearing these substructures were retrieved from the MolPort database by using a SMARTS filter, and submitted to molecular docking against the homology model of LbHsp90N using the same settings as in the first-round virtual screening. Docking poses for the 15% top ranking virtual hits were examined through visual inspection, and 15 molecules (Glb14–Glb28, supplementary Figure S6) were submitted to *in vitro* assays.

### *In vitro* evaluations

Compound selected by virtual screening were tested for their capacity to interact with LbHsp90 and hHsp90β (N-terminal and full-length constructs), as well as for their capacity to impair the viability of the promastigote form of *L. braziliensis* through different *in vitro* approaches. Results are summarised in Table 1 and are described in deeper details below.

**Table 1.** Summary of *in vitro* results obtained in parasite and cell-based assays, as well as against the N-terminal and full-length constructs of LbHsp90 and hHsp90β for the 28 selected compounds.

Ligand	Thermal shift	Cell viability	*IC*_50_ (μM)			*K*_Dapp_ (μM)			
			*L. braziliensis*	Balb-c	Selectivity index	LbHsp90	LbHsp90N	hHsp90β	hHsp90βN
Glb01	No	No	–	–	–	–	–	–	–
Glb02	Yes	No	–	–	–	–	–	–	–
Glb03	No	No	–	–	–	–	–	–	–
Glb04	No	No	–	–	–	–	–	–	–
Glb05	No	No	–	–	–	–	–	–	–
Glb06	No	No	–	–	–	–	–	–	–
Glb07	No	No	–	–	–	–	–	–	–
**Glb08**	Yes	Yes	43 ± 2	75 ± 3	1.7	13 ± 4	17 ± 6	20 ± 5	29 ± 3
Glb09	No	No	–	–	–	–	–	–	–
Glb10	No	No	–	–	–	–	–	–	–
Glb11	Yes	No	–	–	–	–	–	–	–
Glb12	No	No	–	–	–	–	–	–	–
Glb13	No	No	–	–	–	–	–	–	–
Glb14	No	Yes	30 ± 1	>200	>6.7	–	–	–	–
**Glb15**	No	Yes	71 ± 2	>200	>2.8	14 ± 4	13 ± 3	14 ± 6	12 ± 3
Glb16	Yes	Yes	12 ± 2	23 ± 1	1.9	–	–	–	–
Glb17	No	Yes	4 ± 1	58 ± 1	14.5	120 ± 20	100 ± 70	Ambiguous^#^	Ambiguous^#^
Glb18	No	No	–	–	–	–	–	–	–
Glb19	No	No	–	–	–	–	–	–	–
Glb20	No	No	–	–	–	–	–	–	–
Glb21	No	No	–	–	–	–	–	–	–
Glb22	No	No	–	–	–	–	–	–	–
**Glb23**	No	Yes	>100	55 ± 1	<0.5	26 ± 7	20 ± 10	21 ± 9	20 ± 30
Glb24	No	No	–	–	–	–	–	–	–
Glb25	Yes	Yes	>200	11 ± 1	<0.05	7 ± 3	12 ± 2	15 ± 9	14 ± 3
Glb26	No	No	–	–	–	–	–	–	–
Glb27	Yes	Yes	11 ± 1	13 ± 2	1.2	7 ± 1	7 ± 1	5 ± 4	6 ± 2
Glb28	No	Yes	–	–	–	–	–	–	–
GA	Yes	Yes	0.19*	–	–	7.6 ± 0.2**	1.8 ± 0.3**	1.2***	0.77***

Most promising compounds are highlighted in bold.

^#^ Model did not fit to the data. –: not determined (see text for details). *: determined against *L. amazonensis* in reference^36^. **: measured by Tryptophan fluorescence quenching in reference^31^. ***: measured by ITC in reference^35^.

First, the 28 compounds selected *in silico* were preliminarily submitted to DSF analysis to test their capacity to interact with recombinant LbHsp90N and LbHsp90 proteins.

**Figure 1.**
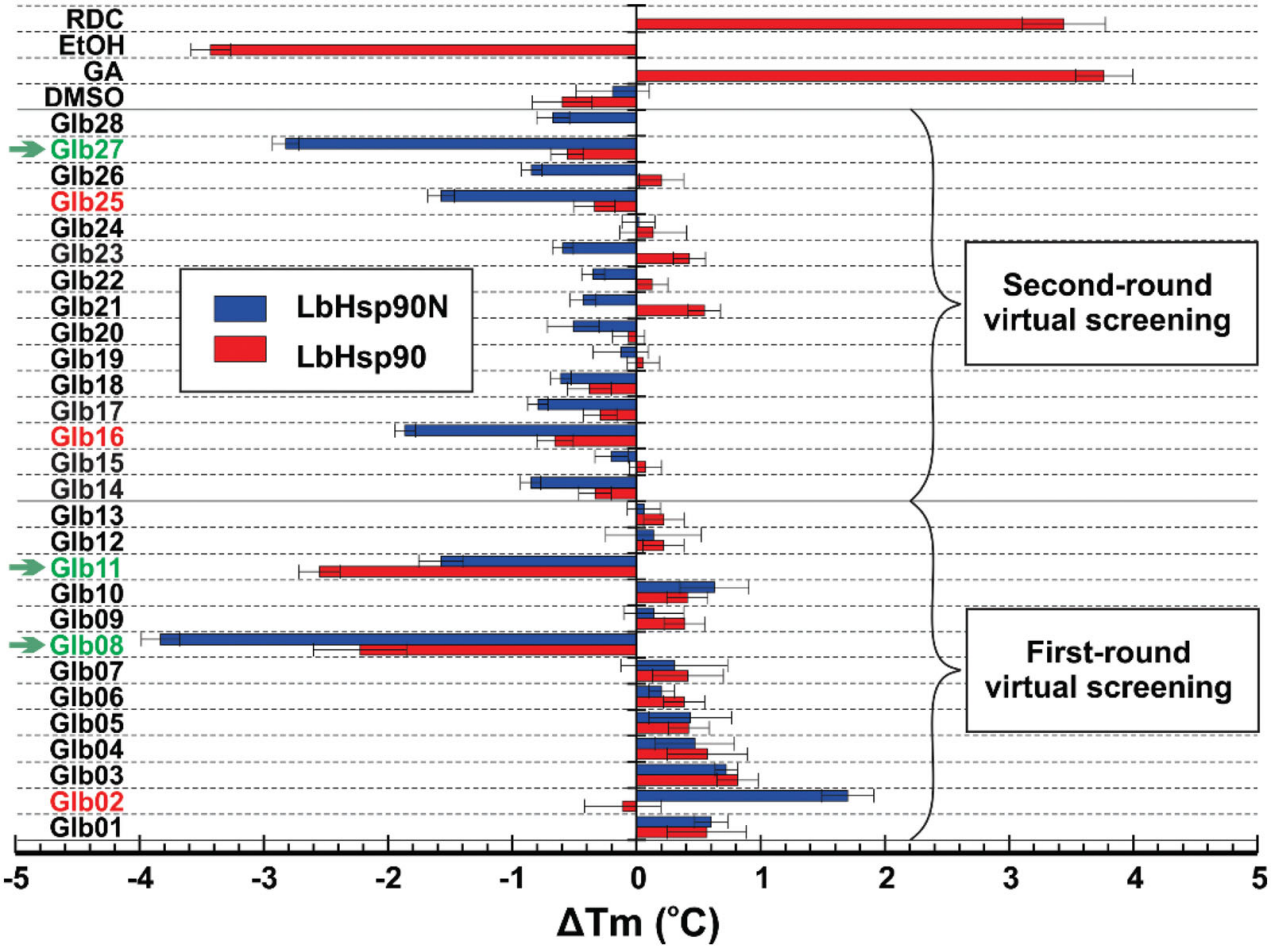
Δ*T_m_* obtained from DSF experiments against LbHsp90 and LbHsp90N recombinant proteins in the presence of known Hsp90 inhibitors RDC and GA, as well as the test set Glb01–Glb28. Compounds marked in green satisfied the inclusion criteria, whereas those marked in red induced borderline Δ*T_m_* changes.

Once obtained the *T_m_* for the above proteins in the presence of each compound, Δ*T_m_* values were calculated as the difference with *T_m_* of isolated proteins (Figure 1). As expected, reference Hsp90 inhibitors RDC and GA led to protein stabilisation with a Δ*T_m_* > 2.0 °C (Figure S7). While testing selected compounds, we established a *T_m_* variation greater or equal to ± 2 °C as inclusion criteria to consider potential Hsp90 binders^21^. Among the 28 tested compounds, Glb02, Glb08, Glb11, Glb16, Glb25 and Glb27 showed positive outcomes by DSF technique (Figure 1). Specifically, comparison of results obtained against LbHsp90N and the full length LbHsp90 clearly highlighted that all compounds might bind preferentially within the N-terminal domain of the protein. Glb11 seems to have additional binding sites than that at the N-terminal, since the Δ*T_m_* against the full length LbHsp90 is higher than that observed for LbHsp90N.

**Figure 2.**
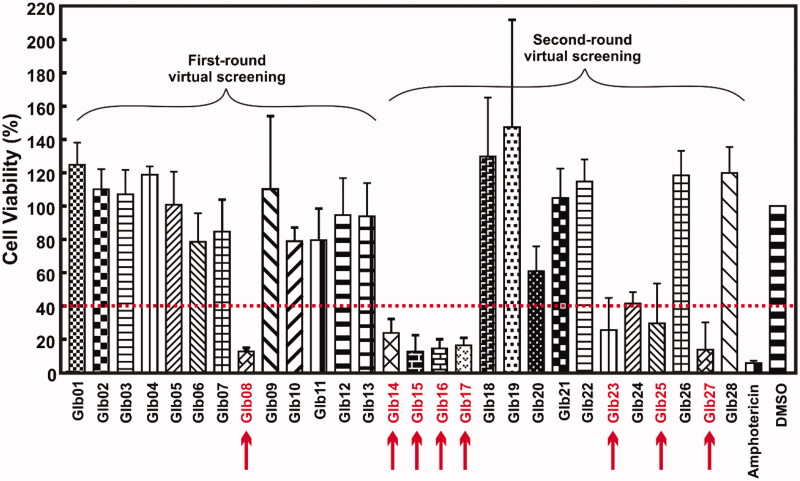
Cell viability experiments. Viability of *L. braziliensis* promastigote cells in the presence of each compound, after 72 h of incubation, assessed by MTT/PMS colorimetric methods. The arrows indicate the compounds impairing more than 50% of cells viability.

Subsequently, we tested the compounds against *L. braziliensis* promastigote cells. Based on the results showed in the Figure 2, compounds Glb08, Glb14, Glb15, Glb16, Glb17, Glb23, Glb25 and Glb27 exhibited leishmanicidal activity as they reduced cell viability more than 60% after 72 h. Amphotericin was used as positive control, showing more than 95% of killing activity (Figure 2).

Eight compounds showed promising results against *L. braziliensis* promastigote cells. However, combining DSF screening with *in vitro* assays led to discontinue compounds Glb02 and Glb11, because they lack leishmanicidal activity although they proved positive in binding to LbHsp90. On the other hand, despite compounds Glb14, Glb15 and Glb17 were not highlighted as potential LbHsp90 binders by DSF, they exhibited leishmanicidal activity towards the parasite and were therefore included in following tests.

**Figure 3.**
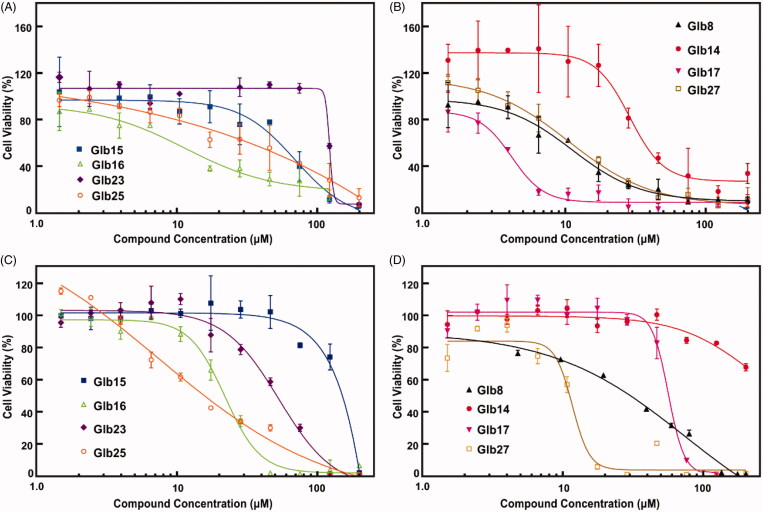
Compounds *IC*_50_ determination against *L. braziliensis* promastigotes cells and their cytotoxic effect against mammalian cells. (A, B) *L. braziliensis* promastigotes cell viability was determined as described in Material and Methods section (Equation (2). (C, D) Compounds cytotoxic activity against Balb/C 3T3 clone A31 mouse fibroblast cells. *IC*_50_ for each compound concentration was fitted by a concentration-dependent equation.

The effect of compounds Glb08, Glb14–Glb17, Glb23, Glb25 and Glb27 on the *L. braziliensis* promastigote cell viability was demonstrated to be concentration-dependent (Figure 3(A,B). Data reported in Table 1 show that Glb08, Glb14, Glb16, Glb17 and Glb27 presented the lowest *IC*_50_ values, with Glb16, Glb17 and Glb27 being the most effective against *L. braziliensis*, whereas Glb15, Glb23 and Glb25 proved active although to a lesser extent.

Given the high degree of structural similarity between LbHsp90 and hHsp90, to monitor the possible selectivity for *L. brazilienzis* with respect to the host, cytotoxicity assays were carried out on Balb-c 3T3 mouse fibroblasts cells. Specifically, compounds showing impairment of *L. braziliensis* promastigotes cell viability (see above) were tested. Results are reported in Figure 3(C,D) and in Table 1 and clearly substantiate that Glb23 and Glb25, endowed with a moderate leishmanicidal activity, also experienced the weakest selectivity. In contrast, Glb27 also showed a weak selectivity for *L. braziliensis* promastigotes. Similarly, Glb08 and Glb16 showed comparable *IC*_50_ values against *L. braziliensis* promastigotes and Balb-c cells, which lead to a low selectivity index. Although showing generally a slightly weaker leishmanicidal activity compared to other compounds, Glb14 and Glb15 seem to not interfere with cell viability of Balb-c cells, which leads to an interesting selectivity index (Table 1). Finally, Glb17 emerged as the most selective compound, also showing the strongest leishmanicidal activity against *L. braziliensis* promastigotes (*IC*_50_ of 4 and 58 µM against *L. braziliensis* promastigotes and Balb-c cells, respectively).

To confirm whether leishmanicidal activity is due to LbHsp90 inhibition, or might be triggered by different mechanisms of action, the direct binding between compounds Glb08, Glb14–Glb17, Glb23, Glb25 and Glb27 and recombinant LbHsp90, hHsp90β and their N-terminal constructs was monitored by fluorescence quenching. This methodology requires that the ligands have a no intrinsic fluorescence (although a low fluorescence might be tolerated) in the same region of the Tryptophan residue (∼300–400 nm). Unfortunately, Glb14 and Glb16 do not match with this requirement, and were not able to be evaluated by the fluorescence quenching assay even after inner filter effect corrections (data not shown).

**Figure 4.**
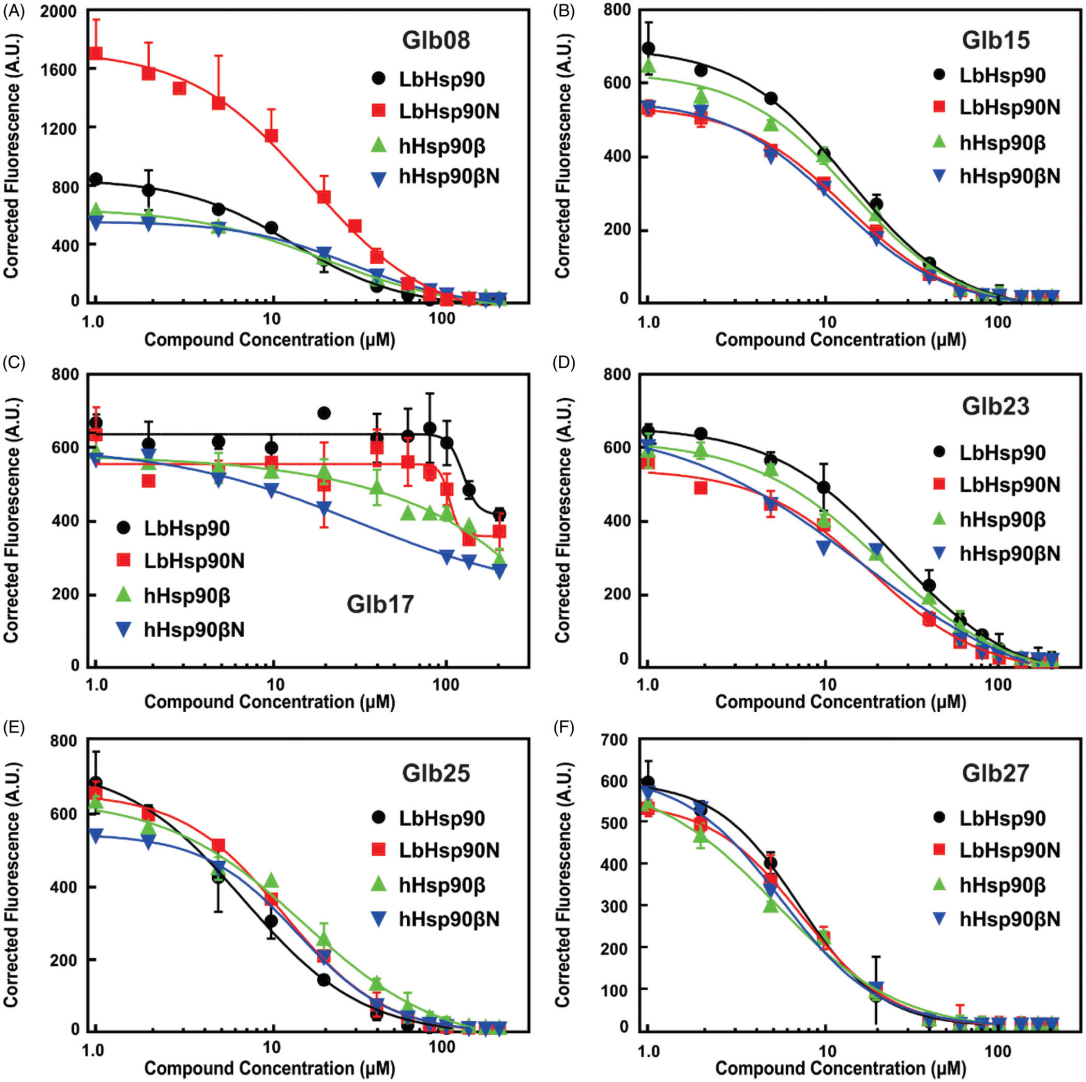
Protein interaction with ligands monitored by the Tryptophan Fluorescence Quenching. The protein fluorescence was monitored in the presence of increasing ligand concentrations and the *K*_Dapp_ was determined as described in Material and Methods section (Equation (3). (A) GLb08, (B) GLb15, (C) Glb17, (D) Glb23, (E) Glb25 and F) Glb27. The continuous line represents the non-linear fitting adjustment with Equation (3) performed by the GrapPrism 6 software.

Figure 4(A) indicates that all tested compounds, with the exception of the Glb17, are able to quench fluorescence of both LbHsp90 and hHsp90β constructs. Notably, *K*_Dapp_ values observed for the interaction with full-length constructions were highly comparable to those monitored for the interaction with the N-terminal domains, which suggests that the preferred binding site of tested compounds is located in the N-terminal region of Hsp90 (Table 1). Glb17 did not induce tryptophan fluorescence quenching neither in the LbHsp90 nor in the hHsp90β constructions (Figure 4(C)), indicating that Glb17 is unable to bind LbHsp90, in agreement with DSF data (Figure 1(B)). Therefore, leishmanicidal activity of Glb17 might be due to the interference with a target that is not LbHsp90, whose identification will be the subject of a further study.

**Figure 5.**
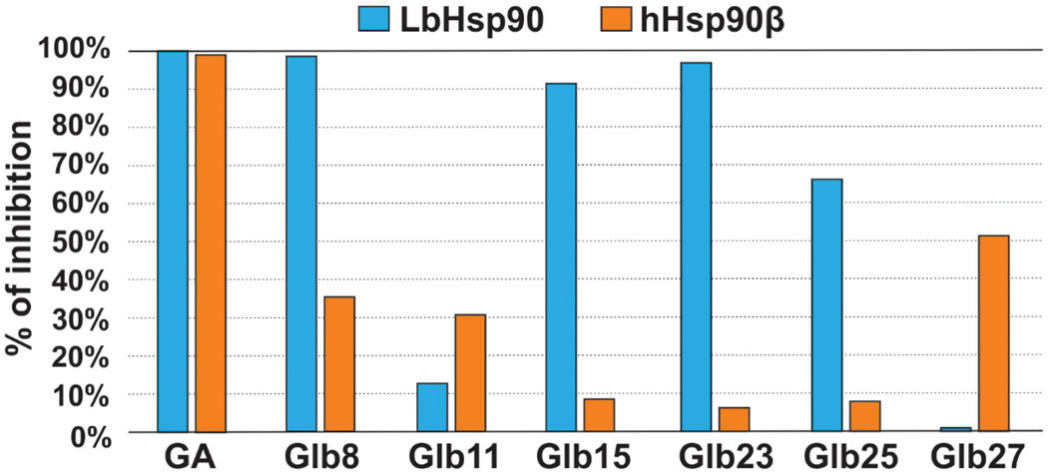
LbHsp90 and hHsp90β ATPase inhibition assay. Percentage of inhibition of GA, Glb8, Glb11, Glb15, Glb23, Glb25 and Glb27 towards LbHsp90 and hHsp90β at 50 µM concentration.

**Figure 6.**
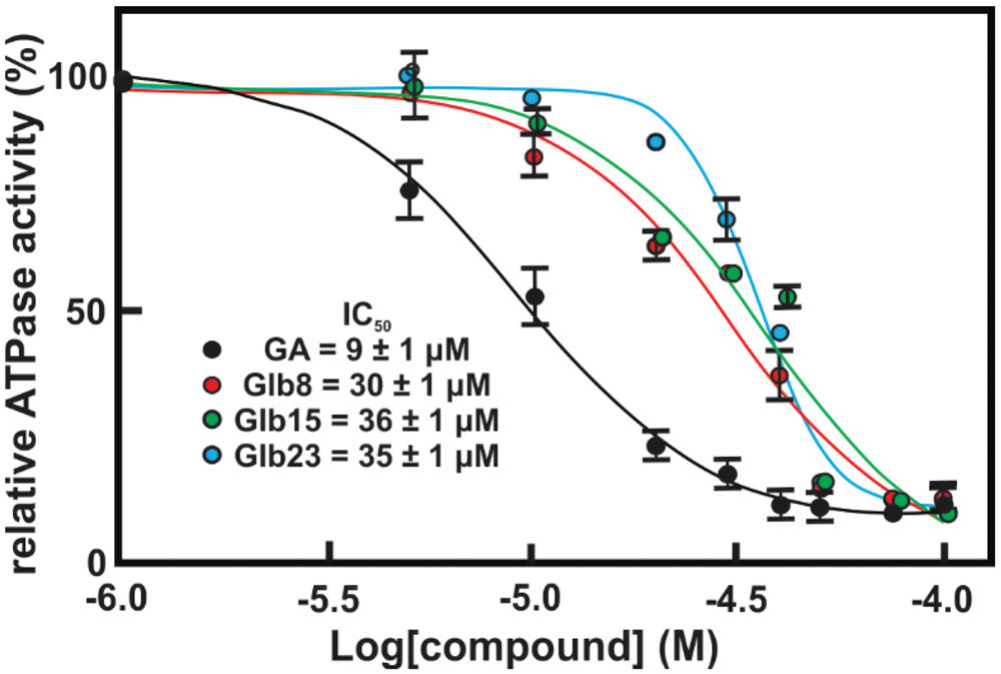
Concentration–response curves of compounds against LbHsp90 ATPase activity inhibition. The ATPase activity inhibition of LbHsp90 was determined as described in Material and Methods section. Concentration-response curve of four compounds, namely (A) Glb08, (B) Glb15, (C) Glb23 and (D) GA. The absolute *IC*_50_ value was determined by nonlinear regression analysis employing GraphPad Prism 6 software.

Since our data suggested that the compounds bind preferentially the N-terminal domain of Hsp90, which exploit its functions through binding and hydrolysing ATP, the effect of compounds Glb08, Glb15, Gl23, Glb25 and Glb27 on the ATPase activity of both recombinant LbHsp90 and hHsp90β was evaluated. All compounds showed a percentage inhibition lower than 60% against hHsp90β, with the only exception of the reference GA that fully inhibits enzyme activity with an *IC*_50_ of 702 nM^11^. On the other hand, compounds Glb08, Glb15 and Glb23 displayed more than 90% inhibition of LbHsp90 at 50 μM (Figure 5) and were submitted to *IC*_50_ determination (Figure 6), showing inhibition at micromolar concentration, and comparable to the value observed for the positive control GA (*IC*_50_ of 9 μM).

Overall, 28 compounds selected by virtual screening (Glb01–Glb28) were tested *in vitro* by different methods. Among them, 8 compounds exhibited leishmanicidal activity (Glb08, Glb14–Glb17, Glb23, Glb25, and Glb27), and 6 were highlighted as potential Hsp90 binders by DSF (Glb08, Glb15, Glb17, Glb23, Glb25, and Glb27). Finally, Glb08, Glb15, and Glb23 were considered as the most promising hits of this series, particularly based on their selective inhibition of ATP hydrolysis in LbHsp90 with respect to the human hHsp90 orthologue.

### Predicted binding modes of Glb08, Glb15 and Glb23 towards LbHsp90N

**Figure 7.**
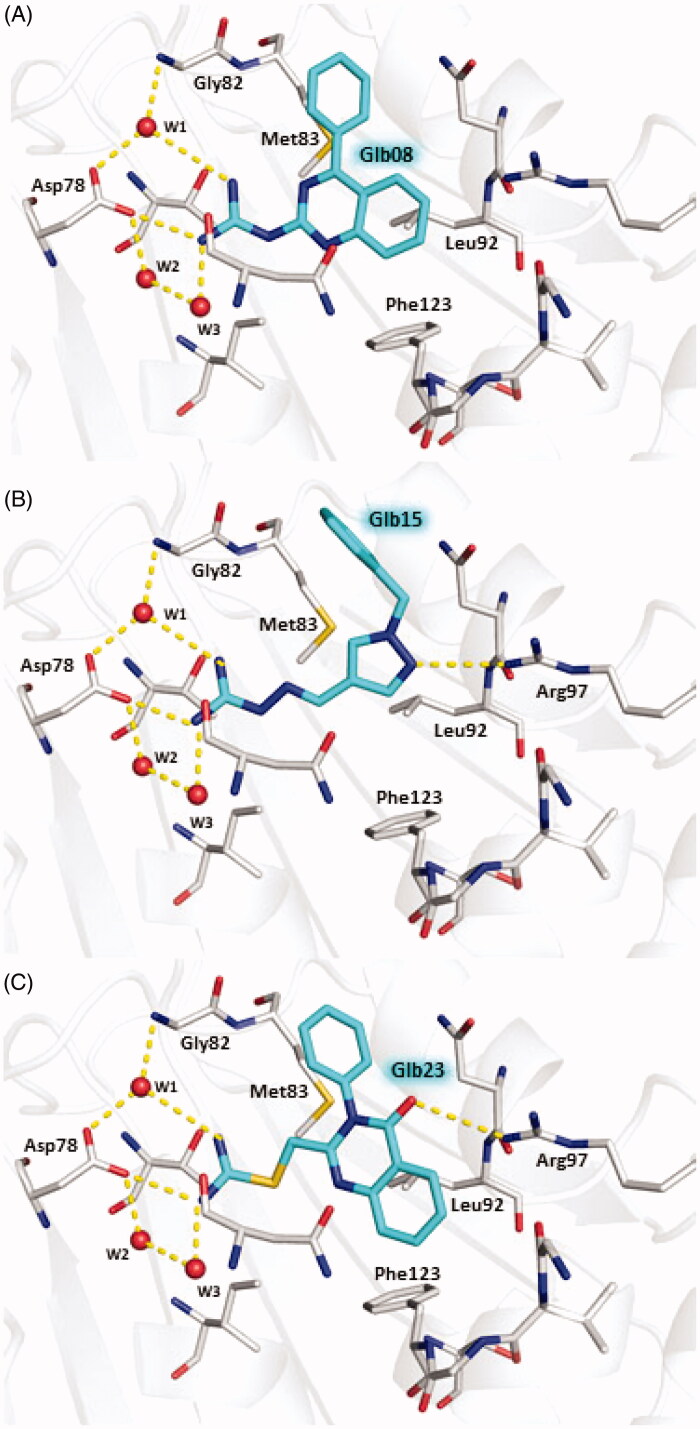
Predicted binding modes of Glb08 (A), Glb15 (B), and Glb23 (C) in LbHsp90N homology model. Glb08, Glb15 and Glb23 are shown in sticks, cyan carbon atoms. LbHsp90 is displayed as grey cartoon with active site residues as sticks, grey carbon atoms (only residues involved in H-bond with the molecules are labelled for clarity). Hydrogen bonds are shown as yellow dashed lines and water molecules as red spheres. Nitrogen atoms are Coloured blue, oxygen red and sulphur yellow. Hydrogen atoms are omitted.

The predicted binding modes of Glb08, Glb15 and Glb23 within the ATP binding site of LbHsp90 are shown in Figure 7. All compounds are able to establish H-bond interactions with Asp78 and the three conserved water molecules W1, W2, and W3 that are known to play a crucial role in binding to the substrate and to some small molecule inhibitors (Figure 7)^35^. A further H-bond interaction bridged by W1 is established by all compounds with the backbone nitrogen of Gly82. The hydrophobic moieties of the three molecules are accommodated inside the hydrophobic cavity lined by Met83, Leu92 and Phe123. According to the predicted binding mode, Glb15 and Glb23 accept an additional H-bond from the side chain of Arg97 (Figure 7(B,C)).

## Discussion

Hsp90 is a well-established target for the treatment of cancer, and is becoming an attractive target also for the therapy of NTD caused by a wide variety of protozoan parasites. In fact, Hsp90 is essential for survival and proliferation of protozoan parasites during their intracellular mammalian stage. Since the ATPase activity executed in the N-terminal domain of Hsp90 is critical for chaperone functions, small molecules able to prevent ATP hydrolysis are expected to inhibit Hsp90, leading to client protein degradation and cell death^37^. In this work, with the ultimate goal to identify hit compounds able to interfere with the replication of *L. braziliensis*, we established a multidisciplinary approach to target LbHsp90N. A structure-based virtual screening combining similarity search with molecular docking simulations was used to screen a commercial library of compounds, and to identify a shortlist of candidate inhibitors of LbHsp90N that were tested *in vitro*. First, DSF and cell viability assays were employed to test the potential of the compounds to bind recombinant LbHsp90, and to impair the survival of *L. braziliensis* promastigotes, respectively. In DSF, both the full length LbHsp90 and its N-terminal domain LbHsp90N were used to monitor whether the compounds might bind within the N-terminal end of the protein endowed with ATP hydrolysis activity.

Among the test set, we found that compounds Glb02, Glb08, Glb11, Glb16, Glb25 and Glb27 induced a change of *T_m_* in at least one LbHsp90 construct, indicating a direct interaction with the target. While some compounds increased protein stability (positive Δ*T_m_* values), others determined a reduction in the thermal stability (negative Δ*T_m_* values) that could be explained by ligand-dependent conformational alterations of the flexible and modular LbHsp90^38^.

Cell viability assay confirmed that above compounds are endowed with leishmanicidal activity, in addition to Glb14, Glb15, Glb17 and Glb23 that were not highlighted by DSF. In contrast, Glb02 and Glb11 did not show leishmanicidal activity against the promastigote form of the parasite. In order to provide deeper insights into the interaction between Glb compounds and LbHsp90, Glb08, Glb16, Glb17, Glb23, Glb25 and Glb27 were also tested by means of tryptophan fluorescence quenching assay. Specifically, ligand interaction with the tryptophan residue located in the ATP binding site of Hsp90 N-terminal domain, which is highly conserved in Hsp90 from multiple species^31^, was monitored.

Notably, all tested compounds proved to bind in the N-terminal region of LbHsp90. However, these molecules proved to bind also hHsp90, generally with similar *K*_Dapp_ as in the LbHsp90. Whether this evidence impacts negatively on the selectivity profile of hit compounds disclosed in this work, this should not be a surprising outcome because of the very high degree of sequence and structural similarity between human and *L. braziliensis* Hsp90.

ATPase activity is essential for the function of Hsp90. The hydrolysis of ATP requires a series of conformational changes, starting from the *apo* state, passing through the closed ATP bonded state to culminate in ATP hydrolysis and release of ADP and P_i_, with Hsp90 returning to its original conformation^39–41^. This suggests that compounds able to mimic or to compete with ATP might behave as inhibitors of Hsp90. By monitoring the compounds effect on Hsp90 ATPase activity, we found that Glb08, Glb15, Gl23, Glb25 and Glb27 did not exhibit extensive inhibition against hHsp90, while Glb08, Glb15 and Glb23 were able to inhibit around 90% of ATPase activity in LbHsp90. *IC*_50_ determination revealed that these molecules inhibit LbHsp90 ATPase activity to a similar extent of the reference inhibitor GA.

It is worth noting that any attempts to characterise the complex between LbHsp90N and selected Glb compounds by X-ray crystallography failed. Atomistic details of the interaction of Glb08, Glb15 and Glb23 to LbHsp90N were provided by molecular modelling. These compounds establish interactions with LbHsp90 Asp78 and Gly82, while non-polar moieties are accommodated inside the hydrophobic cavity lined by Met83, Leu92 and Phe123. Beyond this, Glb15 and Glb23 also performed an additional interaction through Arg97. To evaluate the coherence of these predicted binding modes, they were compared with X-ray crystallographic structures available for LmHsp90N. The complexes of LmHsp90 with ADP (PDB-ID 3U67^21^) with 17-DMAP-geldanamycin (PDB-ID 3Q5J), and with 17-AEP-geldanamycin (PDB-ID 3Q5L) showed that the interactions predicted for our molecules are also relevant for substrates and inhibitors binding to LmHsp90N^42^.

Although the characterisation of the mechanism of action of these compounds would require additional efforts, taken together our results highlighted Glb08, Glb15, and Glb23 as confirmed inhibitors of LbHsp90 that are worth of further development. These molecules might serve as source of inspiration in the design of further generation of LbHsp90 small molecule inhibitors with improved potency, selectivity, and drug-like features.

## Conclusions

In this work, we used a multidisciplinary approach boosted by computational screening to identify a number of small molecules able to impair the viability of *L. braziliensis* promastigotes, to bind LbHsp90, or to inhibit ATPase activity exerted by LbHsp90N. Particularly, our molecules bear a guanidine-like pharmacophore that – to the best of our knowledge – is not included in any known Hsp90 inhibitors targeting the N-terminal domain reported to date. These molecules could be used as a starting point to design further generations of LbHsp90 inhibitors with improved activity, drug-like properties, and selectivity.

## Supplementary Material

Supplemental Material
