# Supplementary material for: Discovery of small molecule inhibitors of Leishmania braziliensis Hsp90 chaperone
Source: J Enzyme Inhib Med Chem. 2020 Feb 12;35(1):639–49. doi: 10.1080/14756366.2020.1726342 (PMC7034072; doi:10.1080/14756366.2020.1726342)
Supplement: Supplemental Material [file IENZ_A_1726342_SM7037.pdf]

Supporting Information

Discovery of small molecule inhibitors of *Leishmania Braziliensis* Hsp90 chaperone

Fernanda A. H. Batista[a1], Sérgio L. Ramos Jr[a], Giusy Tassone[b], Andrei Leitão[c], Carlos A. Montanari[c], Maurizio Botta[b,d,e], Mattia Mori[b*], Júlio C. Borges[a]

[a] São Carlos Institute of Chemistry (IQSC), University of São Paulo (USP), São Carlos, SP, Brazil.

[b] Department of Biotechnology, Chemistry and Pharmacy - Department of Excellence 2018-2022, University of Siena, via Aldo Moro, 2, 53100 Siena (SI), Italy.

[c] Medicinal Chemistry Group (NEQUIMED), IQSC-USP, São Carlos, SP, Brazil.

[d] Lead Discovery Siena S.r.., Castelnuovo Berardenga, Siena, Italy.

[e] Sbarro Institute for Cancer Research and Molecular Medicine, Center for Biotechnology, College of Science and Technology, Temple University, Philadelphia, PA, United States of America.

```
LbHsp90N     MTETFAFQAEINQLMSLIINTFYSNKEIFLRELISNASDACDKIRYQSLTDPSVLGEETH (60)
LmHsp90N     MTETFAFQAEINQLMSLIINTFYSNKEIFLRELISNASDACDKIRYQSLTDPSVLGESPR (60)
             *********************************************************. :

LbHsp90N     LRVRVVPDKANKTLTVEDNGIGMTKADLVNNLGTIARSGTKAFMEALEAGGDMSMIGQFG (120)
LmHsp90N     LCIRVVPDKENKTLTVEDNGIGMTKADLVNNLGTIARSGTKAFMEALEAGGDMSMIGQFG (120)
             * :****** **************************************************

LbHsp90N     VGFYSAYLVADRVTVVSKNNSDEAYVWESSAGGTFTITSVPESDMKRGTRITLHLKEDQQ (180)
LmHsp90N     VGFYSAYLVADRVTVTSKNNSDESYVWESSAGGTFTITSTPESDMKRGTRITLHLKEDQM (180)
             ***************.*******:***************.*******************

LbHsp90N     EYLEERRVKELIKKHSEFIGYDIELMVEKTAEKEVTDEDEE (221)
LmHsp90N     EYLEPRRLKELIKKHSEFIGYDIELMVEKTTEKEVTDEDEE (221)
             **** **:**********************:**********
```

Figure S1. Sequence alignment between LbHsp90N and LmHsp90N. Residues involved in ATP binding are indicated by a red dot above the residue.

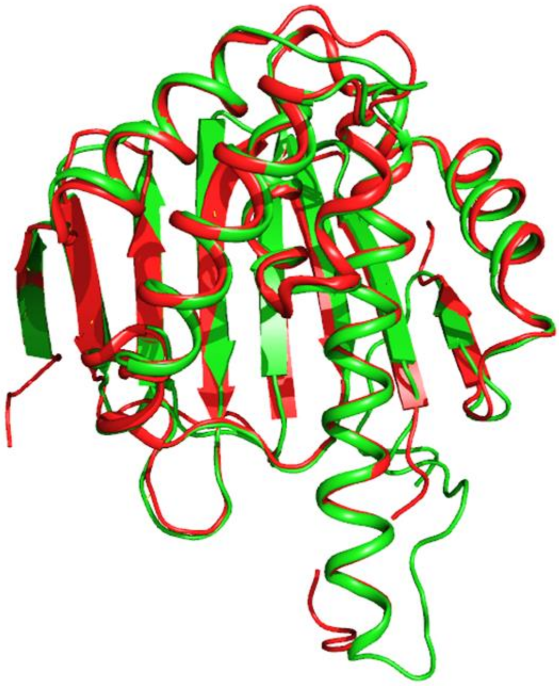

Figure S2. Structural superposition between the template, LmHsp90N (PDB ID 3U67 (21)) and the model obtained through the homology modeling displayed as red and green cartoon, respectively.

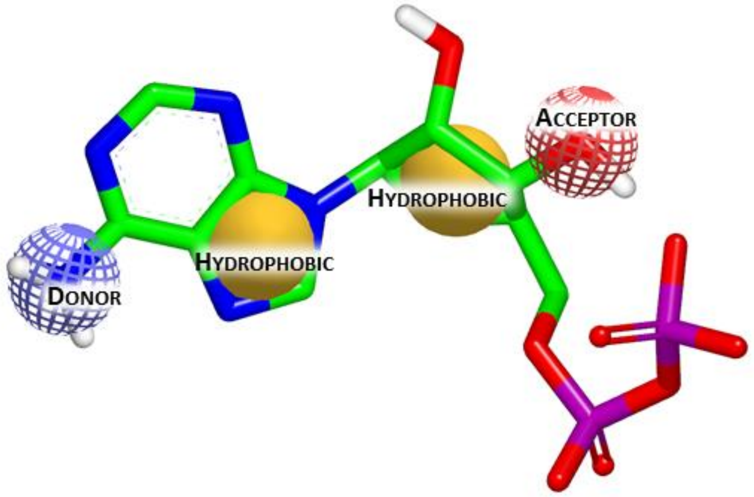

Figure S3. The main features of the ADP query are highlighted as spheres. Query features are coloured as follows: hydrophobic = yellow; acceptor = red; donor = blue.

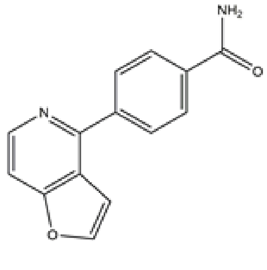

Glb01

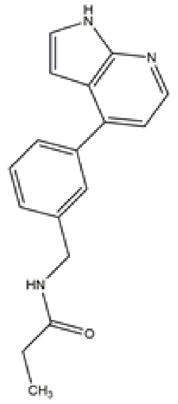

Glb02

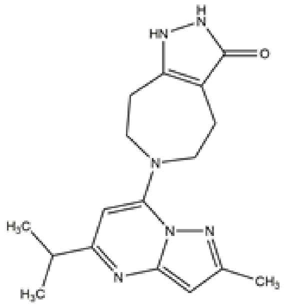

Glb03

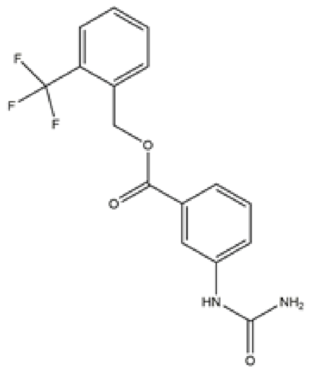

Glb04

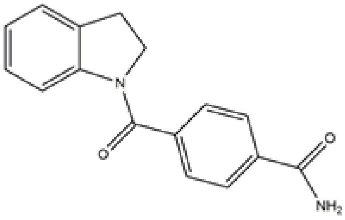

Glb05

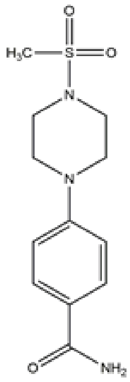

Glb06

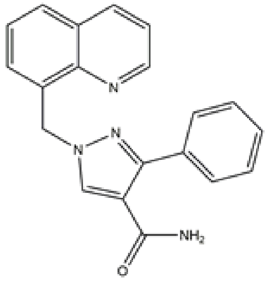

Glb07

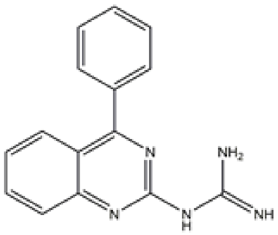

Glb08

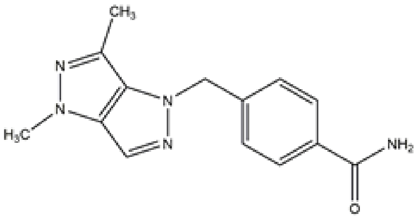

Glb09

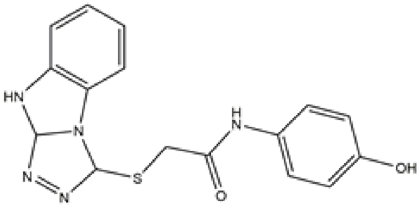

Glb10

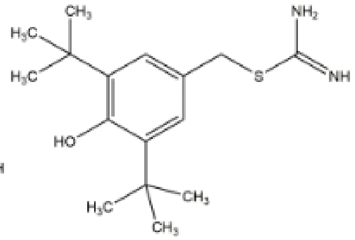

Glb11

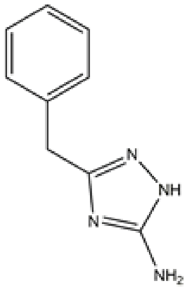

Glb12

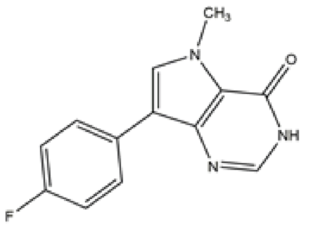

Glb13

Figure S4. Chemical structure of compounds identified in the first round of virtual screening against LbHsp90N.

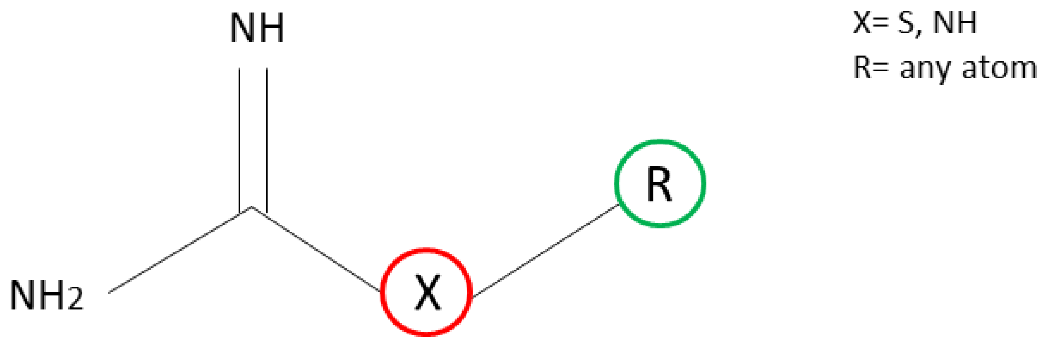

Figure S5. 2D representation of Glb08 and Glb11 substructure. X can be either a sulphur or an amino group whereas R can be any chemical group.

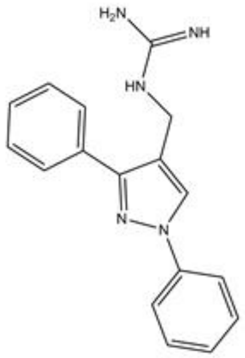

Glb14

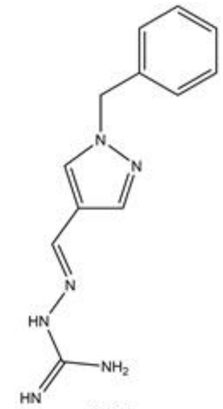

Glb15

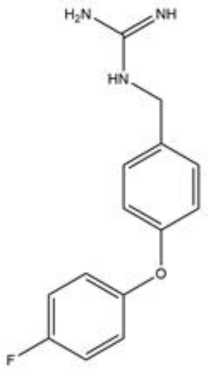

Glb16

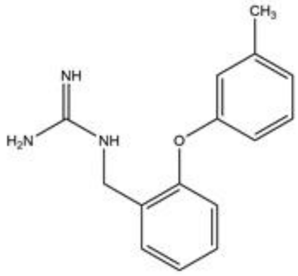

Glb17

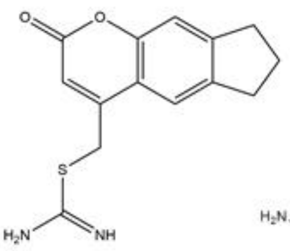

Glb18

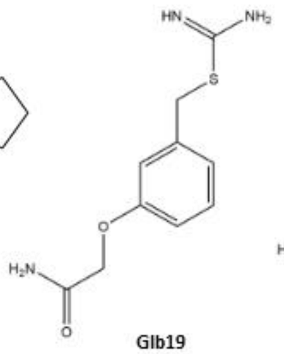

Glb19

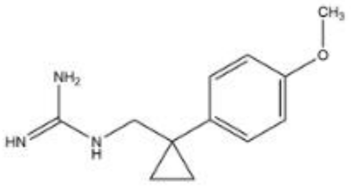

Glb20

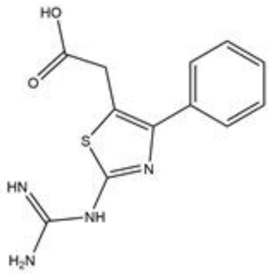

Glb21

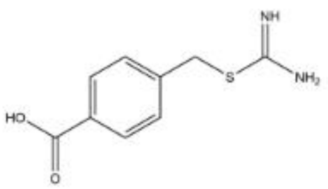

Glb22

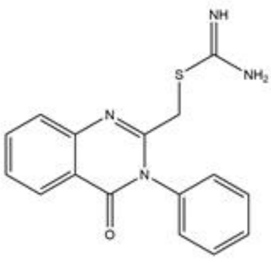

Glb23

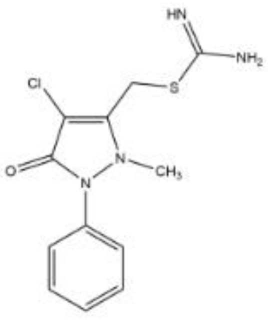

Glb24

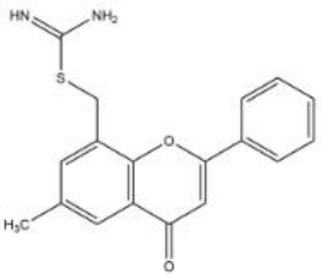

Glb25

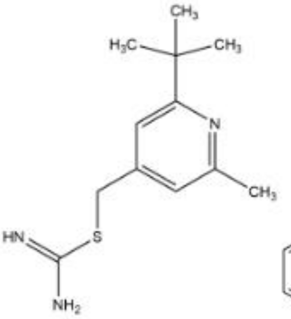

Glb26

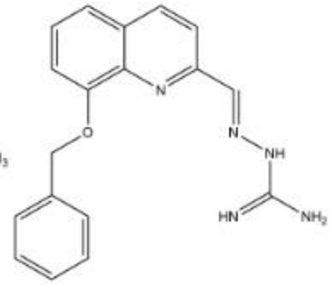

Glb27

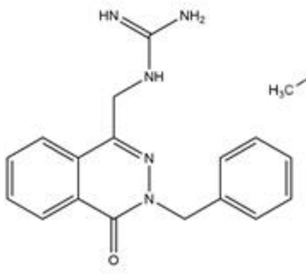

Glb28

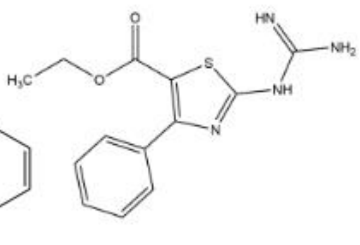

Glb29

Figure S6. Chemical structure of compounds identified in the second round of virtual screening against LbHsp90N.

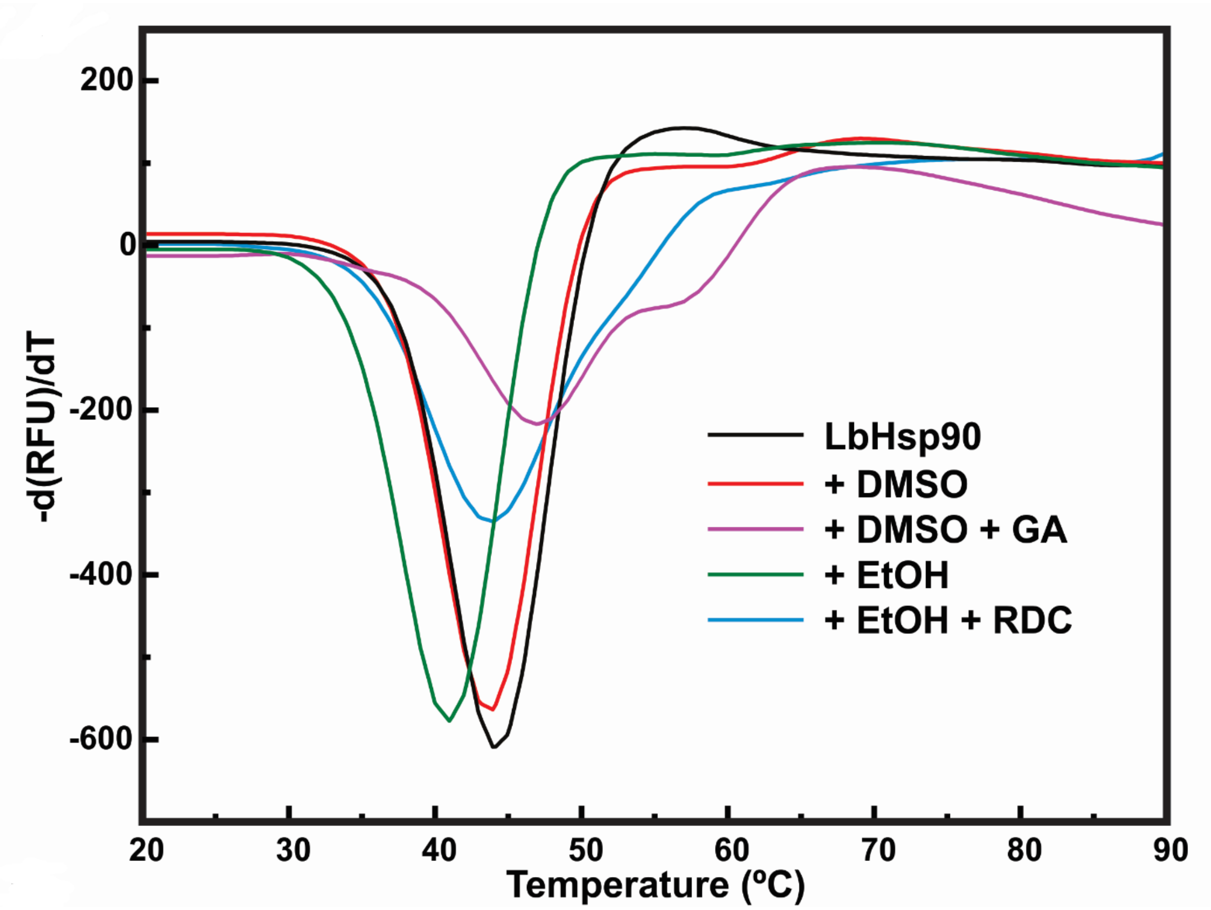

Figure S7. DSF experiments for LbHsp90 and LbHsp90N recombinant proteins. Representative DSF experiments for LbHsp90 in the absence and presence of GA or RDC. DMSO and ethanol were used as vehicles for those compounds. The curves represent the first derivative of the raw data of a representative set of DSF experiments for LbHsp90 with known ligands of Hsp90 N-terminal domains, such as GA and RDC. The plot represents the first derivative of the raw data showing a transition centered at around 45 ºC, which is correlated to its N-terminal domain as previously reported by differential scanning calorimetry. GA and RDC induced a shift of the curves to higher temperatures when compared to the LbHsp90 in the presence of DMSO and ethanol, which were used as vehicles for GA and RDC respectively. The melting temperature (Tm) for each condition were determined as the peak of the first derivative and each value is an average of three curves.
